# Randomized comparison of power Doppler ultrasonography-guided core-needle biopsy with open surgical biopsy for the characterization of lymphadenopathies in patients with suspected lymphoma

**DOI:** 10.1007/s00277-017-2926-9

**Published:** 2017-01-27

**Authors:** Novella Pugliese, M. Di Perna, I. Cozzolino, G. Ciancia, G. Pettinato, P. Zeppa, V. Varone, S. Masone, C. Cerchione, R. Della Pepa, L. Simeone, C. Giordano, V. Martinelli, C. Salvatore, F. Pane, M. Picardi

**Affiliations:** 1 Department of Clinical Medicine and Surgery, Federico II University Medical School, Naples, Via S. Pansini 5, 80131 Naples, Italy; 2 Department of Advanced Biomedical Sciences, Federico II University Medical School, Naples, Naples, Italy; 3 Department of Medicine and Surgery, University Medical School, Salerno, Salerno, Italy; 4 Department of Economics, University of Molise, Campobasso, Italy

**Keywords:** Lymphoma, Power Doppler ultrasonography, Core-needle cutting biopsy

## Abstract

The sensitivity of lymph node core-needle biopsy under imaging guidance requires validation. We employed power Doppler ultrasonography (PDUS) to select the lymph node most suspected of malignancy and to histologically characterize it through the use of large cutting needle. Institutional review board approval and informed consent were obtained for this randomized clinical trial. In a single center between 1 January 2009 and 31 December 2015, patients with lymph node enlargement suspected for lymphoma were randomly assigned (1:1) to biopsy with either standard surgery or PDUS-guided 16-gauge modified Menghini needle. The primary endpoint was the superiority of sensitivity for the diagnosis of malignancy for core-needle cutting biopsy (CNCB). Secondary endpoints were times to biopsy, complications, and costs. A total of 376 patients were randomized into the two arms and received allocated biopsy. However, four patients undergoing CNCB were excluded for inadequate samples; thus, 372 patients were analyzed. Sensitivity for the detection of malignancy was significantly better for PDUS-guided CNCB [98.8%; 95% confidence interval (CI), 95.9–99.9] than standard biopsy (88.7%; 95% CI, 82.9–93; *P* < 0.001). For all secondary endpoints, the comparison was significantly disadvantageous for conventional approach. In particular, estimated cost per biopsy performed with standard surgery was 24-fold higher compared with that performed with CNCB. The presence of satellite enlarged reactive and/or necrotic lymph nodes may impair the success of an open surgical biopsy (OSB). PDUS and CNCB with adequate gauge are diagnostic tools that enable effective, safe, fast, and low-cost routine biopsy for patients with suspected lymphoma, avoiding psychological and physical pain of an unnecessary surgical intervention.

## Introduction

In the case of clinical suspicion of lymphoma, the histological examination of lymphadenopathy is essential for defining a correct diagnosis and for developing a proper treatment plan [1]. An open surgical biopsy (OSB) is still the “gold standard,” owing to the large amount of tissue obtained [2]. Preoperative evaluation includes (1) a careful and through physical examination, i.e., palpation of superficial lymph node regions performed by a physician experienced in the management of patients with lymphoma; (2) gray-scale ultrasonography scans (US), i.e., a technology that is readily available in clinical practice and is considered to provide sufficient information for selecting the node to be biopsied [1, 2]; and (3) computed tomography (CT), performed to strengthen the suspicion of lymphoma [2]. However, the possible presence of enlarged reactive or necrotic lymph nodes and/or of nonpalpable but histologically significant malignant lymph nodes may impair the success of an OSB. Another limitation is mostly related to patients whose conditions may be too unstable for undergoing general anesthesia and surgical intervention [3]. Thus, a study that has value to decide the primary interventionist diagnostic tool for suspected lymphoma is a clinically important topic. New mini-invasive approaches to this procedure based on imaging-guided methods are now available.

The introduction of the new generation of ultrasonographic and biopsy needle devices, which already have been proven valuable in the management of patients with lymphoma in our cancer center [4–8], provides the opportunity to develop effective combined diagnostic strategy. The modern US instruments merge tissue harmonic compound, which generates an image from multiple imaging lines that strike the target from different angles [9], with power Doppler (PD) which allows the study of the angioarchitecture of lymph node tissue [5, 6, 8, 10]. Neoplastic angiogenesis such as vessel proliferation (endothelial cell migration and proliferation) and abnormal vascularization (tube formation with stenosis, occlusion, and/or dilation and/or arteriovenous shunts) is recognized as being critical for B cell lymphoma pathogenesis [11, 12]. Power Doppler ultrasonography (PDUS) equipment detects fine flow signals, mimicking an angiography of microvascular intranodal network. The result is a high-resolution quality examination that allows better detection of both superficial and deep-seated malignant lymphadenopathies compared with results obtained with gray-scale US [13]. Regarding biopsy needle devices, the latest Menghini needles have ultrathin sharpened cannula with trocar stylet and automatic aspiration with tiny battery-powered vacuum [14]. These characteristics make particularly effective the needle devices with large gauge [15]. Under PDUS guidance, the tip of cutting needle can be careful positioning into the most significant target, obtaining histological suction of the core of nodal lesion [14–16]. Nevertheless, few clear indications for performing such procedure are available. The Lugano classification for initial evaluation, staging, and response assessment of Hodgkin and non-Hodgkin lymphoma recommend core-needle biopsy when surgical intervention is not possible and to document relapse [2]. However, the existing guidelines are not evidence based, a uniform program for optimal imaging guidance is lacking, and the characteristics of biopsy needle, i.e., gauge, length, tip configurations, and sampling mechanisms, are still a matter of opinion among experts [1, 14, 16]. Thus, this approach requires validation with randomized studies.

Our trial was intended to test the efficacy of PDUS-guided core-needle cutting biopsy (CNCB) compared with OSB as first-line diagnostic approach for pathologic lymphadenopathies in patients with clinical suspicion of lymphoma. The primary endpoint of the study was the sensitivity for diagnosis of malignancy for each of the two interventionist methods, i.e., percutaneous biopsy by using modified Menghini needle under modern US guidance and standard excisional biopsy. Additional endpoints were times to biopsy, rates of biopsy-related complications, and costs.

## Materials and methods

### Trial design and participants

Included patients were randomly assigned at 1:1 allocation ratio to receive lymph node biopsy by using one of two methods, OSB (standard group) or PDUS-guided CNCB (core-needle group).

Patients were required to meet the following eligibility criteria: (a) age ≥14 years, (b) lymph node enlargement clinically suspected for lymphoma, and (c) indication to perform nodal biopsy. Patients affected by Epstein-Barr virus, cytomegalovirus, herpes simplex virus, rubella, toxoplasma, or tuberculosis infection, as well as abnormalities of coagulation tests were excluded.

This was a single center study. Eligible patients were registered at the Hematology Division Office of the “Federico II” University of Naples, where the trial was designed and approved by the local Institutional Review Board in the early 2008 (10 January 2008; number of registration, 140/2008).

### Interventions

#### Standard group

In the standard group, all biopsy-related procedures were performed by surgeons experienced in lymph node resection. The patients underwent physical examination and gray-scale US, of whom findings were sufficient to account for the region to be biopsied according to conventional methods [17]. At surgeon’s discretion, biopsy was directed to the most superficial and/or largest lymph node. In a day hospital regimen or as inpatients, and under local or general anesthesia (according to the type of intervention scheduled), the lymph nodes were harvested through skin crease incision obtained by free-hand methods. Superficial lymphadenopathy was removed by means of excisional biopsy. Mini-cervicotomy or mediastinotomy were used for removing lymphadenopathy in the anterosuperior mediastinum, and abdominal and pelvic lymphadenopathies were removed by means of laparotomy.

#### Core-needle group

In the core-needle group, all biopsy-related procedures were performed by two members of the hematology staff (N. Pugliese and *M. Picardi*, with more than 10 years of experience with interventionist PDUS) [4, 5]. The lymph node to undergone CNCB was determined by PDUS assessment as already reported [8]. In particular, baseline US exploration of all superficial, anterosuperior mediastinum (clavicular, supra-aortic, and prevascular regions), and abdominal and pelvic lymph node areas was carried out. Then, any abnormal [for size (long axis ≥ 2 cm), round shape, hilus absent, and/or hypoechoic parenchyma] lymph node underwent power Doppler examination in accordance with methods already described [5, 6, 8], using a scanner (iU22; Philips Health-care, Bothell, Wash) equipped with tissue harmonic compound technology (SonoCT; Philips), power Doppler sonography, and 5–1 MHz (C5-1 curvilinear; Philips) and 9–3 MHz (L9-3 linear; Philips) broadband probes. The main criterion to select the node to be biopsied was the hypervascularization, i.e., intranodal arterial vessels with high-resistive index value (>0.6) [6, 8]. All CNCB were carried out under US guidance with a puncture adaptor, an aseptic technique (sterile cover of the probe and sterile gel), and cutaneous anesthesia, using a 16-gauge diameter modified Menghini needle 150 mm in length with automatic aspiration (Biomol® HS-Hospital; Rome, Italy).

### Reference standard

The reference standard for lymph node involvement was histopathologic examination. It was performed in a single pathology unit by at least three expert hematopathologists (I. Cozzolino, G. Ciancia, G. Pettinato, P. Zeppa, and/or V. Varone, with more than 10 years of experience with hematopathological analysis) [5]. Lymph node samples were routinely fixed in formalin and embedded in paraffin (FFEP). The histologic sections were stained according to standard methods (hematoxylin and eosin, and Giemsa). All cases of lymphoma were diagnosed by a combination of morphologic, immunohistochemical and/or molecular analyses and were classified according to the current WHO criteria [1]. Immunophenotyping was carried out in FFEP slides with antibodies recognizing CD3, CD4, CD8, CD5, CD10, CD15, CD20, CD23, CD30, CD45RB, CD56, CD79a, bcl-2, bcl-6, cyclin D1, PAX-5, Mum-1, Ki-67, ALK-1, and TdT. *Bcl-2*, *Myc*, *Cyclin D1*, and *MALT-1* gene translocations were evaluated by fluorescent in situ hybridization analysis in FFPE slides using commercially available kits, whenever deemed necessary. B or T cell clonality was also investigated by polymerase chain reaction. Epithelial metastatic tumors were identified by monoclonal antibodies to cytokeratin.

Overall, biopsies were categorized as positive for malignancy (samples containing adequate number of cells with morphologic atypia and evidence of monoclonality), negative for malignancy (samples containing adequate number of cells with no evidence of malignancy), or inadequate (specimens too small to confirm or rule out malignancy). Patients classified as having histologic results negative for malignancy underwent strict follow-up by clinicians for the following months, in order to discovery a malignant disease undetected at first biopsy.

In 50 patients of the experimental arm, the biopsy specimens of nodal tissue were studied by the three operators: each one was blinded to the patient’s clinical condition and to the histologic results of the other hematopathologists (interobserver reproducibility) [15].

### Primary and secondary outcomes

The sensitivity for each arm was defined as the ratio of patients who showed lymph node positive for malignancy at first biopsy compared with the total number of patients with malignancy. In addition, the negative predictive value was defined as the ratio of patients with lymph node negative for malignancies at first biopsy compared to the total number of patients with negative results for malignancy during the follow-up. The likelihood ratio of a negative test was also calculated (1 minus sensitivity divided by specificity).

The waiting time for the performance of biopsy was calculated as the number of days elapsed between indication to lymph node biopsy and the execution of the procedure itself.

After biopsy, patients were strictly monitored in order to look for procedure-related complications. Outpatients were kept under observation for 1 h and were discharged if there were no signs or symptoms suggestive of a significant complication. All patients were encouraged to contact their physicians if they developed symptoms after leaving hospital.

Cost analysis for biopsy procedures was performed by adopting the perspective of the National Healthcare System. Cost calculations for PDUS-guided CNCB were based on the tariffs in the Nomenclature for Outpatient Care, provided by the Italian National Healthcare System (http://www.arsan.campania.it/documents/10157/01088316-4824-4c7e-8671-1418af8f3af7). The costs of OSB were calculated according to the diagnosis-related group tariffs that are currently used to fund in patient health services in Italy (http://www.eumed.it/drg/tariffe_drg.asp).

### Sample size

We tested the hypothesis that histological yield obtained with PDUS-guided CNCB resulted in a higher sensitivity than OSB, owing to a more significant lymph node tissue biopsied (i.e., the viable core of malignant lesion was exactly removed). Based on previous studies, we estimated a sensitivity rate at standard biopsy of 78% [5] and at PDUS-guided CNCB of 96.5% [14, 18]; hence, a certain number of patients could be underdiagnosed with OSB approach. To detect more than 10% sensitivity improvement (for the superiority test), 332 patients were needed, when using a two-sided type I error of 5% and 99% statistical power. Assuming a dropout rate of 10%, we set a final sample size of at least 183 patients in each group.

### Randomization

Random allocation sequence was carried out by using a computerized system (generated by the study statistician on the basis of the procedure outlined elsewhere) [19]. It was based on a minimization method in which patients were assigned to the two study groups while ensuring equal distribution on the basis of sex, age, presence and type of systemic symptoms (i.e. fever, sweating, and weight loss) and sites of lymph node enlargement at baseline clinical evaluation.

Patients were asked to sign a consent form before randomization, according to the requirements of the Helsinki declaration.

### Statistical analysis

For the statistical evaluations, the *χ* ^2^ test was performed to compare proportions for clinical and histological characteristics and complication rate, and the *t* test was used to compare the quantitative variables of clinical characteristics, costs, and waiting times to biopsy between the two groups. *P* values less than 0.05 were considered to indicate a significant difference.

Asymptotic 95% confidence intervals for kappa statistic (to assess the level of agreement in diagnostic opinion among all three hematopathologists for the 50 samples of the core of nodal tissue) were computed according to Fleiss et al. [20].

## Results

### Participants and recruitment

**Fig. 1:**
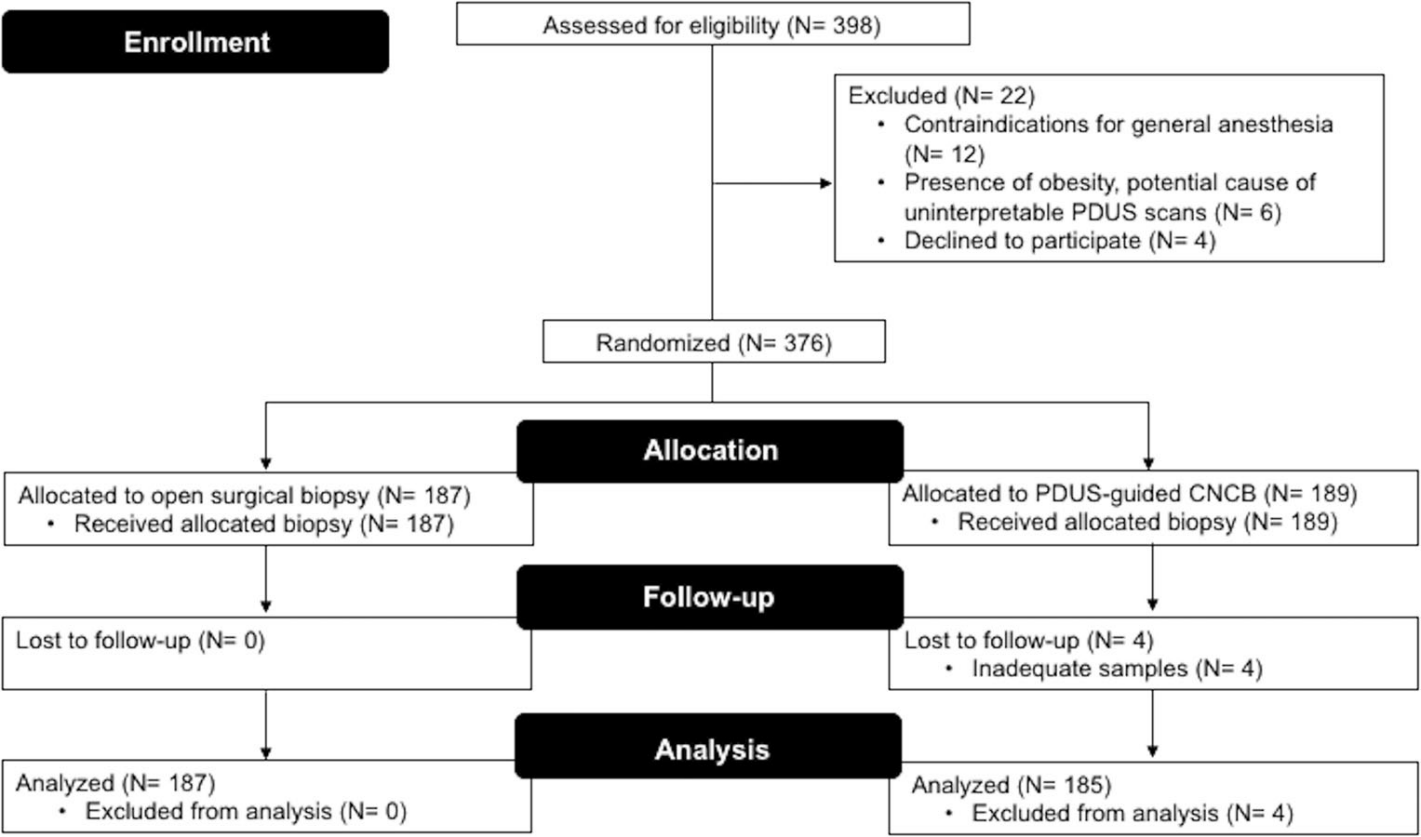
Flowchart shows patient selection and follow-up during the study (CONSORT). *PDUS* = power Doppler ultrasonography

Between 1 January 2009 and 31 December 2015, 376 patients were randomly assigned either to standard group (*N* = 187) or core-needle group (*N* = 189). All randomized patients received allocated biopsy intervention. However, four patients (2.1%) undergoing PDUS-guided CNCB were excluded for inadequate samples (thereafter, these cases underwent an OSB). No other patient was lost to follow-up, nor did any withdraw their consent to participate in the study when a second biopsy was clinically indicated during monitoring. Thus, a total of 372 patients was analyzed for the primary endpoint (standard group, *N* = 187; core-needle group, *N* = 185). Twenty-two patients (5.5%) failed during screening. A common reason for exclusion was contraindications for general anesthesia (*N* = 12). Other reasons were the presence of obesity, potential cause of uninterpretable PDUS scans for deep-seated lymph nodes (*N* = 6), and refused to participate (*N* = 4). A consolidated standard of reporting trials’ (CONSORT) diagram summarizes the study in Figure 1.

Patients in both groups were well-balanced with respect to clinical characteristics, in particular symptoms suspected for lymphoma and nodal sites involved at baseline evaluation (Table 1).

**Table 1:** Baseline characteristics of patients in the two study groups

	Standard group	Core-needle group	*P* value
Total patients	187	185	
Sex			
Male	98 (52.4)	86 (46.5)	0.25
Female	89 (47.6)	99 (53.5)	
Age, years			
Median, (range)	46 (18–79)	42 (17–76)	0.61
Symptoms			
Fever	33 (17.6)	31 (16.8)	0.82
Sweat	24 (12.8)	25 (13.5)	0.84
Weight loss	27 (14.4)	26 (14.1)	0.91
Site of clinically suspected lymphadenopathies			
Cervical	93 (49.7)	90 (48.6)	0.83
Axillary/pectoral	41 (21.9)	39 (21.1)	0.84
Antero-superior mediastinum	4 (2.1)	3 (1.6)	0.71
Inguinal	28 (15)	30 (16.2)	0.74
Abdomen-pelvic	21 (11.2)	23 (12.4)	0.72

Note: unless otherwise indicated, data are number of patients, with percentage in parentheses

### Power Doppler ultrasonographic and core-needle features

The average time required for PDUS examination and core-needle biopsy was 40 min (range, 30–50 min). Sites of biopsied lymph nodes were superficial in 140 cases (vs. 160 cases in the standard group) and deep-seated (abdominal or pelvic regions) in 45 cases (vs. 27 cases in the standard group, *P* = 0.02). For each core-needle biopsy, a median of 2 needle passes (range, 1–4) into the nodal tissue was made. Length of core-needle specimens varied from 15 to 70 mm (median, 32 mm). Median-estimated volume of acquired tissue was 185 mm^3^ with a range of 92–430 mm^3^ (vs. a median volume of 1458 mm^3^ with a range of 312–5678 mm^3^, in the standard group). The number of tests (i.e., staining and/or molecular analyses) performed by pathology on core-needle tissue and surgical excisional biopsy was similar in the two study groups.

Interobserver reproducibility of histological assessments of the cores of nodal tissue among the three pathologists had a kappa score of 0.916 (95% CI: 0.756–1.07). Of the 50 samples tested for reproducibility, 49 (98%) were classified identically by the three observers.

### Histology

Of the 187 patients undergoing OSB, 149 (80%) cases had lymph nodes positive for malignancy, and 38 (20%) had lymph nodes negative for malignancy (described as benign lymphoid hyperplasia in 37 cases, and sarcoidosis in one case, with steato-fibrotic and/or necrotic changes in 17 of the cases).

Of the 185 patients undergoing PDUS-guided CNCB (all with adequate specimens), 172 (93%) cases had lymph nodes positive for malignancy, and 13 (7%) had lymph nodes negative for malignancy (benign lymphoid hyperplasia in 10 patients, Kikuchi-Fujimoto disease in two patients, and sarcoidosis in one patient; Table 2).

**Table 2:** Histologic diagnosis on lymph node biopsy in the two study groups

	Standard group (*N* = 187)	Core-needle group (*N* = 185)
B cell neoplasms	84 (44.9)	97 (52.4)
Diffuse large B cell lymphoma	32 (17.1)	38 (20.5)
Follicular lymphoma	25 (13.4)	23 (12.4)
CLL/SLL^a^	16 (8.6)	18 (9.7)
Mantle cell lymphoma	7 (3.7)	12 (6.5)
Nodal marginal zone lymphoma	3 (1.6)	5 (2.7)
Primary mediastinal (thymic) large B cell lymphoma	1 (0.5)	1 (0.5)
Hodgkin lymphoma	38 (20.3)	46 (24.9)
Nodular sclerosis	25 (13.4)	30 (16.2)
Mixed cellularity	9 (4.8)	11 (5.9)
Nodular lymphocyte predominant	2 (1.1)	2 (1.1)
Lymphocyte-rich	1 (0.5)	1 (0.5)
Lymphocyte-depleted	1 (0.5)	2 (1.1)
T cell neoplasms	4 (2.1)	8 (4.3)
Anaplastic large cell lymphoma, ALK-positive	2 (1.1)	4 (2.2)
T cell lymphoblastic leukemia/lymphoma	1 (0.5)	2 (1.1)
Peripheral T cell lymphoma	1 (0.5)	1 (0.5)
Anaplastic large cell lymphoma, ALK-negative	–	1 (0.5)
Metastatic carcinoma	23 (12.3)	21 (11.4)
Nonmalignant findings	38 (20.3)	13 (7)
True-negative	19 (10.1)	11 (5.9)
Benign lymphoid hyperplasia	18 (9.6)	8 (4.3)
Sarcoidosis	1 (0.5)	1 (0.5)
Kikuchi-Fujimoto disease	–	2 (1.1)
False-negative	19 (10.1)	2 (1.1)
Benign lymphoid hyperplasia^b^	19 (10.1)	2 (1.1)

Note: unless otherwise indicated, data are number of patients, with percentage in parentheses

*ALK* anaplastic lymphoma kinase

^a^Chronic lymphocytic leukemia/small lymphocytic lymphoma

^b^With steato-fibrotic and/or necrotic changes in 17 of the cases

Overall, the 51 patients with lymph nodes negative for malignancy (defined as reactive or inflammatory) were observed for a median follow-up of 10 months (range, 1–24 months). During the follow-up, for 19 of 38 patients in the standard group, the clinicians required a second lymph node biopsy, and a malignancy was finally detected. The second biopsy, which was performed after a median of 5 months (range, 1–9 months) from the first biopsy, demonstrated lymphoma in 16 patients (five diffuse large B cell lymphomas, three grade 1 follicular lymphomas, two small lymphocytic lymphomas, four Hodgkin lymphomas, one mantle cell lymphoma, and one nodal marginal zone lymphoma) and metastatic carcinoma in three patients (Table 3). In contrast, two of the 13 patients who had had diagnosis of a benign lesion at the first biopsy in the core-needle group required a second biopsy (open surgical intervention in both cases) after 6 and 8 months, respectively. Histologic examination showed a malignancy in both cases (one grade 1 follicular lymphoma and one small lymphocytic lymphoma) (Table 3).

**Table 3:** Findings in the patients who underwent a second lymph node biopsy (all open surgical biopsies) in the two study groups

Patient No.	No. of months between the two biopsies	Biopsy site		Sample volume (mm^3^)		Histologic diagnosis	
		First	Second	First	Second	First	Second
1	2	Cervical	Axillary	1597	2154	Benign hyperplasia^b^	Diffuse large B cell lymphoma
2	4	Inguinal	Mesenteric	1460	2092	Benign hyperplasia^b^	Diffuse large B cell lymphoma
3	3	Cervical	Supraclavicular	3200	4230	Benign hyperplasia^b^	Diffuse large B cell lymphoma
4	5	Supraclavicular	Axillary	1539	2129	Benign hyperplasia	Diffuse large B cell lymphoma
5	6	Inguinal	Iliac	5148	2766	Benign hyperplasia	Diffuse large B cell lymphoma
6	1	Cervical	Supraclavicular	2860	1769	Benign hyperplasia^b^	Nodular sclerosis—HL
7	3	Cervical	Cervical	4512	2870	Benign hyperplasia^b^	Nodular sclerosis—HL
8	3	Axillary	Supraclavicular	1955	2350	Benign hyperplasia^b^	Nodular sclerosis—HL
9	4	Inguinal	Cervical	2766	2020	Benign hyperplasia	Nodular sclerosis—HL
10	5	Cervical	Cervical	2030	1980	Benign hyperplasia	Follicular lymphoma Grade I
11	6	Cervical	Axillary	3240	2563	Benign hyperplasia	Follicular lymphoma Grade I
12	7	Inguinal	Inguinal	1780	1201	Benign hyperplasia^b^	Follicular lymphoma Grade I
13	6	Cervical	Supraclavicular	673	1251	Benign hyperplasia^b^	CLL/SLL
14	8	Cervical	Inguinal	1840	2560	Benign hyperplasia^b^	CLL/SLL
15	5	Cervical	Supraclavicular	790	1300	Benign hyperplasia	Mantle cell lymphoma
16	9	Supraclavicular	Axillary	1578	3410	Benign hyperplasia	Nodal marginal zone lymphoma
17	1	Cervical	Supraclavicular	4370	2531	Benign hyperplasia	Metastatic carcinoma
18	2	Inguinal	Inguinal	3594	1589	Benign hyperplasia	Metastatic carcinoma
19	5	Cervical	Supraclavicular	1737	2010	Benign hyperplasia^b^	Metastatic carcinoma
20^a^	6	Supraclavicular	Supraclavicular	230	2130	Benign hyperplasia	Follicular lymphoma Grade I
21^a^	8	Inguinal	Cervical	310	1867	Benign hyperplasia^b^	CLL/SLL

Note: Unless otherwise indicated, data are number of patients, with percentage in parentheses

*HL* Hodgkin lymphoma, *CLL/SLL* Chronic lymphocytic leukemia/small lymphocytic lymphoma

^a^Patients #20 and #21 had received power Doppler ultrasonography-guided core-needle cutting biopsy as first lymph node biopsy

^b^With intranodal steato-fibrotic and necrotic changes

The definitive histological findings for each case in the two groups are shown in Tables 2 and 3. Overall, the majority of patients were suffering from lymphomas (B cell non Hodgkin lymphoma, 195 cases; Hodgkin lymphoma, 88 cases; T cell non Hodgkin lymphoma, 12 cases; and metastatic carcinoma, 47 cases).

### Accuracy in identifying malignancy

The sensitivity rate of lymph node malignant status was 88.7% [95% confidence interval (CI): 82.9–93] for OSB (149 of 168 patients with lymph node positive for malignancy were identified) with a false negative rate of 10.2% (19 of 168 patients with lymph node positive for malignancy were not identified). By contrast, the sensitivity rate of lymph node malignant status was 98.8% (95% CI: 95.9–99.9) for PDUS-guided CNCB (172 of 174 patients with lymph node positive for malignancy were identified) with a false negative rate of 1.1% (i.e., 2 of 174 patients with lymph node positive for malignancy were not identified). Therefore, the study objective to show superiority of PDUS-guided CNCB versus OSB was achieved, being the sensitivity rate of experimental approach significantly higher than the standard approach (*P* < 0.001; Table 4).

**Table 4:** Accuracy of standard biopsy and PDUS-guided CNCB for the diagnosis of malignant lymph nodes

	Standard group (*N* = 187)	Core-needle group (*N* = 185)	*P* value
Sensitivity			
*N*	149/168	172/174	0.0001
%	88.7	98.8	
95% CI	82.9–93.0	95.9–99.9	
False-negative			
*N* (%)	19 (10.2)	2 (1.1)	0.0001
Negative predictive value			
*N*	19/38	11/13	0.014
%	50	84.6	
95% CI	33.4–66.6	54.5–98.1	
Negative likelihood ratio			
value	0.11	0.01	
95% CI	0.07–0.17	0.00–0.05	

*CNCB* core-needle cutting biopsy, *CI* confidence interval

Noteworthy, the sensitivity rate of lymph nodes positive for lymphoma was 98.7% (95% CI: 95.4–99.8) for PDUS-guided CNCB versus 88.7% (95% CI: 82.3–93.4) for OSB (*P <* 0.001). The negative predictive value was 54.3% (95% CI: 36.6–71.2) for OSB and 84.6% (95% CI: 54.5–98.1) for PDUS-guided CNCB (*P* = 0.05). The negative likelihood ratio was 0.11 (95% CI: 0.07–0.18) for OSB and 0.01 (95% CI: 0.00–0.05) for PDUS-guided CNCB, confirming the value of the PDUS-guided CNCB for detecting lymphoma.

### Waiting time to biopsy

The median waiting time for performance of interventionist procedure (from biopsy indication to perform itself) in the core-needle group was 4 days (range, 1–10 days). By contrast, it was 16 days with a range of 5–34 days in the standard group (*P* < 0.001).

### Procedure-related complications

Overall, 42 patients, which were in the standard group, underwent biopsy (cervical-clavicular, 17 cases; mediastinum compartments, 4 cases; abdomen-pelvis, 21 cases) under general anesthesia, with an average hospitalization of 2.5 days. All other patients underwent biopsy in a day surgery or outpatient regimen under local anesthesia.

Patients who received standard biopsy had significantly more pain, numbness or paresthesia, larger scars, lymphorrhea, and wound infection than patients who underwent PDUS-guided CNCB (Table 5).

**Table 5:** Biopsy-related complications in the two study groups

	Standard group (*N* = 187)	Core-needle group (*N* = 185)	*P* value
Pain on operated site^a^			
No	46 (24.6)	130 (70.3)	<0.0001
Yes, mild and transient	57 (30.5)	39 (21.1)	0.038
Yes, continuous	84 (44.9)	16 (8.6)	<0.0001
Numbness on operated site			
No	42 (22.5)	134 (72.4)	<0.0001
Yes	145 (77.5)	51 (27.6)	
Swelling on operated site			
No	50 (26.7)	162 (77.6)	0.0008
Yes	137 (73.3)	23 (12.4)	
Esthetic appearance of biopsy scar^b^			
Absent	–	185 (100)	<0.0001
Acceptable	85 (45.5)	–	
Unpleasant	102 (54.5)	–	
Hematoma^c^			
No	177 (94.6)	179 (96.8)	0.31
Yes	10 (5.4)	6 (3.2)	
Lymphorrhoea			
No	178 (85.2)	185 (100)	0.0025
Yes	9 (4.8)	–	
Wound infection			
No	175 (93.6)	185 (100)	0.0005
Yes	12 (6.4)	–	

Note: unless otherwise indicated, data are number of patients, with percentages in parentheses

^a^Postoperative pain was evaluated as absent, mild (not requiring analgesia), or continuous (requiring analgesia)

^b^As judge by the patients themselves 1 month after biopsy

^c^Temporary hemorrhage, spontaneously resolved

### Cost analysis

The total cost of the biopsy program was much lower for the core-needle group than that for the standard group. By using Italian values for direct costs of interventionist procedures, the cost for one OSB was €10,393 for major surgery and €3056 for minor surgery, whereas it was €171 for one PDUS-guided CNCB (including the complete US assessment of superficial and deep-seated lymph node areas). If the cost of additional surgical biopsies in the 19 patients (false negative results) of the standard group and in the two patients (false negative results) of the core-needle group is considered, the total cost of lymph node biopsy with standard approach was approximately 25-fold higher than that with PDUS-guided CNCB (*P* < 0.001; Table 6). C. Salvatore wrote the section devoted to cost analysis and produced Table 6.

**Table 6:** Cost analysis of biopsy procedures

Examinations and costs	Standard group (*N* = 187 patients)	Core-needle group (*N* = 185 patients)
Total no. of biopsy procedures	187	185
Unitary cost for biopsy (€)		
Major surgery^a^	10,393	–
Minor surgery^b^	3056	–
Complete US assessment of superficial and deep-seated nodal areas (€)	–	88
US-guided core-needle cutting biopsy (€)	–	83
Average cost of biopsy procedure per patient (€)	4115	171
Total cost of additional surgical biopsies due to false-negative results (€)^c^	153,445	6112
Total cost of biopsy program (€)	923,016	37,747

^a^Major surgery includes mini-cervicotomy, mediastinotomy and laparotomic bioptic procedure

^b^Minor surgery includes excisional biopsy of superficial lymph nodes

^c^Total cost of additional surgical biopsies for the four patients randomized in the core-needle group, but excluded for inadequate samples, was 41,572 €

## Discussion

Routine biopsy of lymphadenopathies by using core-needle under imaging guidance in patients with suspicion of lymphoma is controversial [1, 2]. Usually, such procedures are reserved for lymph nodes that are accessible only with surgical risk or for critical ill patients, and in case of relapse [2]. Most studies on this issue were retrospective, included an imaging support based on traditional radiological tools (such as gray-scale US and CT, which study mostly morphological characteristics, i.e., the dimensional features of lymph node, not distinguishing between viable tumor and inflammation, necrosis and/or fibrosis), and have tested the role of small (≥18-gauge) needle devices with obsolete configurations [16]. Thus, it is reasonable to investigate a front line diagnostic combination of new generation imaging equipment and technologically refined cutting needle with large gauge [15].

Our randomized study was an examination of two different interventionist approaches, one surgery-driven (standard arm) and the other one hematology-driven (experimental arm), in patients with lymphadenopathies clinically suspected for lymphoma. Traditionally, whole lymph nodes are resected when it is necessary to determine whether a lymphadenopathy is lymphoma or some other conditions, such as metastases of a nonhematological tumor [1, 2]. In our trial, the entire decision making process for biopsy in the standard arm was left to the surgeon’s discretion: the selection of the node to be biopsied was based on physical examination and gray-scale US [17]. In the daily diagnostic service of our surgery unit, as in others [21], power Doppler ultrasonographic technology was limited in its availability for routine clinical practice. In the experimental arm, the selection of the node to be biopsied and biopsy itself were exclusively based on the expertise of hematologists. In fact, the hematological unit kept a modern US equipment available and had it run by experienced operators, who were members of the hematological staff trained in diagnostic PDUS [4, 5]. The goal of the study was to maintain optimal accuracy of the diagnostic work-up of lymphadenopathies, while avoiding psychological and physical pain of an unnecessary surgical intervention.

**Fig. 2:**
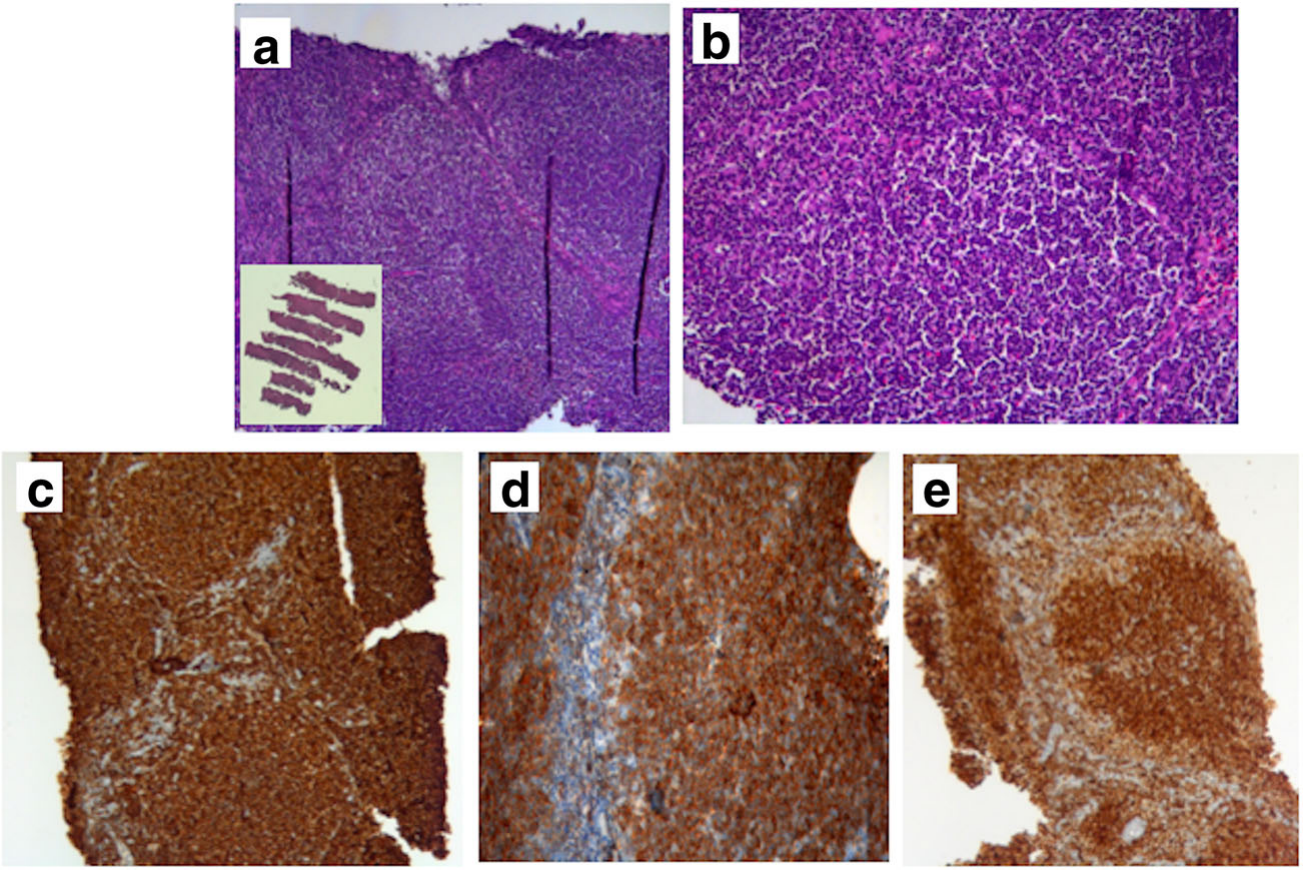
**a** Inset: low-power image (H&E, ×1) of a core-needle biopsy specimen obtained from a right iliac lymph node: the core-needles reveal large follicular nodules closely packed with a back-to-back arrangement (H&E, ×20). **b** The neoplastic lymphoid follicles are composed of a uniform, small size, cell population (H&E, ×40). **c**, **d**, **e** The immunohistochemical stain strongly highlights CD20 (**c**), CD10 (**d**), and BCL-2 (**e**) (ABC, ×40). These samples are large enough to preserve tissue architecture and to assess the diagnosis of follicular lymphoma

**Fig. 3:**
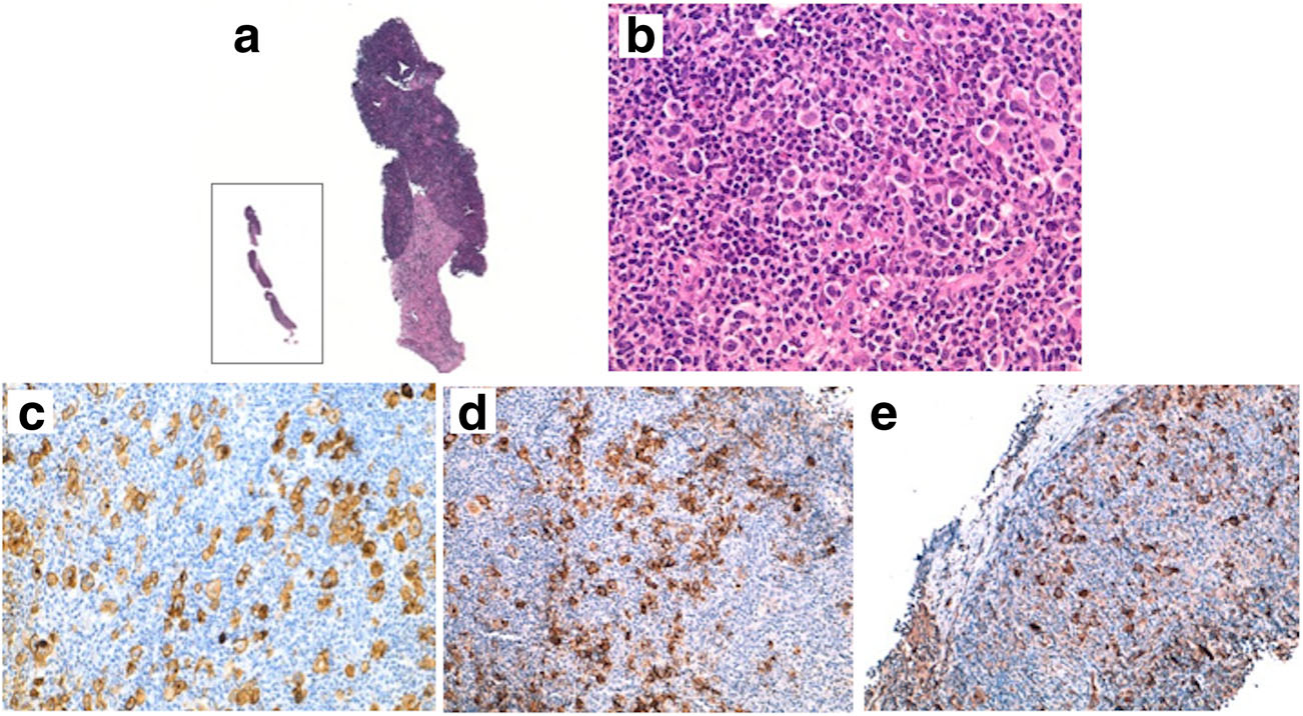
**a** Inset: low-power image (H&E, ×1) of a core-needle biopsy specimen obtained from a right latero-cervical lymph node: the core-needle appears fragmented due to an obvious fibrosis (H&E, ×5). **b** Higher power views show several Reed-Sternberg cells (H&E, ×40). The Reed-Sternberg cells are CD30 (**c**), CD15 (**d**), and fascin (**e**) positive (ABC, ×40). These samples are large enough to preserve tissue architecture and to assess the diagnosis of nodular sclerosis classical Hodgkin lymphoma

The primary endpoint in this trial, a greater sensitivity with the experimental approach, was proven being the comparison with standard approach significantly advantageous for PDUS-guided CNCB. The number of cases in which a definite diagnosis of malignancy could not be established at first biopsy was almost 8 times higher on standard tissue specimens than core-needle material. As a consequence, the clinicians recommended a re-biopsy (OSB) significantly more often in the standard group than experimental group, also considering the four patients with inadequate samples randomized in the core-needle group (19 vs. 6 cases, respectively; *P* = 0.006). Not all lymph nodes may be involved by the main disease entity. There is a risk of removing satellite reactive lymph nodes, thus missing the primary diagnosis of a malignant disease present in another node, which is sometimes deeper seated or even seated in a different anatomic area. An affected lymph node may also undergo necrosis and/or steato-fibrotic changes, which could avert the pathologist from the correct diagnosis. These are all potential sources of inaccuracy in standard excisional biopsy [5]. In our study, PDUS technology accurately selected the most suspected target, imaged all nodal lesion clearly, and simultaneously monitored the entire puncture process (in both superficial and deep-seated regions). The cutting needle had a diameter of 1.6 mm with ultrathinner tip and wall, and powered automatic suction. Although the tissue volume obtained by CNCB was smaller than OSB, the experimental method provided enough tissue for architectural-morphologic pattern assessment, immunohistochemical staining, and/or molecular testing (Figs. 2 and 3) [1].

For all secondary endpoints in this trial, the comparison was significantly disadvantageous for excisional biopsy. Compared with PDUS-guided CNCB, standard approach had significantly more waiting time to allocated interventionist procedure, considerably higher amounts of biopsy-related complications (analgesia required for postoperative pain was about 5-fold higher), and extraordinarily higher costs for the National Healthcare System (performance of one biopsy was 24-fold more expensive with standard approach).

Our study suffers from three major limitations. First, this trial was conducted in one single center. Therefore, studies from other institutions are needed to assess (1) interobserver and interequipment PDUS variability; (2) core-needle specimen quality reproducibility, e.g., tissue harvested, size and preservation; and (3) concordance by pathologists in diagnosing and subtyping lymphoma on core-needle material. Second, a bias error could have been committed due to a more accurate selection of nodal target to be biopsied in the core-needle group leading the study toward a better sensitivity for experimental arm than standard arm. A factor that may have a strong influence to explain such bias is the high specialization (derived from long and extensive experience) [4–8] of hematology team to identify the right lymph node and to biopsy it, as compared to the surgeons. Finally, the rate of failure with PDUS-guided CNCB was 1.6% (6/376 patients randomly allocated to core-needle biopsy procedure). In four patients (those with inadequate samples), stiffened tissue of nodular sclerosis Hodgkin lymphoma (documented at re-biopsy) which was seated in subclavicular area (a particular hindered region) led to the sampling error of CNCB. For the two false negative results (benign lymphoid hyperplasia), the final diagnosis (at second biopsy) was conclusive for small lymphocytic indolent non-Hodgkin lymphoma, suggesting that in some instances, there is a need of a large amount of lymph node tissue for correct histological assessment.

To the best of our knowledge, this study is the first to compare in a randomized fashion the sensitivity of imaging-guided CNCB and OSB in detecting lymphoma. Under optimal study conditions (avoiding patients with obesity), with modern US equipment and an experienced operator, core-needle biopsy is a reliable and cost-effective diagnostic procedure [22]. Histological patterns of lymphoma are recognizable in core material and are useful in diagnosing and subtyping according to the current *WHO classification of tumors of haematopoietic and lymphoid tissues* [1, 15]. A 16-gauge cutting needle is recommended, and at least two passes yielding two tissue cores, with total length of 30–60 mm should be taken. CNCB is less traumatic and well tolerated by patients. It should be recommended as first-line procedure, for both superficial and deep-seated lymph nodes, for patients with a suspected lymphoma, and not merely for patients with poor medical condition when surgical intervention is not possible or to document relapse.
